# Firefly algorithm based WSN-IoT security enhancement with machine learning for intrusion detection

**DOI:** 10.1038/s41598-023-50554-x

**Published:** 2024-01-02

**Authors:** M. Karthikeyan, D. Manimegalai, Karthikeyan RajaGopal

**Affiliations:** 1 Centre for Advanced Wireless Integrated Technology, Chennai Institute of Technology, Chennai, India; 2 Department of Electrical and Electronics Engineering, RajaLakshmi Engineering College, Thandalam, India; 3 Centre for Nonlinear System, Chennai Institute of Technology, Chennai, India

**Keywords:** Energy science and technology, Engineering

## Abstract

A Wireless Sensor Network (WSN) aided by the Internet of Things (IoT) is a collaborative system of WSN systems and IoT networks are work to exchange, gather, and handle data. The primary objective of this collaboration is to enhance data analysis and automation to facilitate improved decision-making. Securing IoT with the assistance of WSN necessitates the implementation of protective measures to confirm the safety and reliability of the interconnected WSN and IoT components. This research significantly advances the current state of the art in IoT and WSN security by synergistically harnessing the potential of machine learning and the Firefly Algorithm. The contributions of this work are twofold: firstly, the proposed FA-ML technique exhibits an exceptional capability to enhance intrusion detection accuracy within the WSN-IoT landscape. Secondly, the amalgamation of the Firefly Algorithm and machine learning introduces a novel dimension to the domain of security-oriented optimization techniques. The implications of this research resonate across various sectors, ranging from critical infrastructure protection to industrial automation and beyond, where safeguarding the integrity of interconnected systems are of paramount importance. The amalgamation of cutting-edge machine learning and bio-inspired algorithms marks a pivotal step forward in crafting robust and intelligent security measures for the evolving landscape of IoT-driven technologies. For intrusion detection in the WSN-IoT, the FA-ML method employs a support vector machine (SVM) machine model for classification with parameter tuning accomplished using a Grey Wolf Optimizer (GWO) algorithm. The experimental evaluation is simulated using NSL-KDD Dataset, revealing the remarkable enhancement of the FA-ML technique, achieving a maximum accuracy of 99.34%. In comparison, the KNN-PSO and XGBoost models achieved lower accuracies of 96.42% and 95.36%, respectively. The findings validate the potential of the FA-ML technique as an active security solution for WSN-IoT systems, harnessing the power of machine learning and the Firefly Algorithm to bolster intrusion detection capabilities.

## Introduction

The IoT denotes to a network of interconnected devices communicating over the internet^1^. Within this IoT framework, Wireless Sensor Networks (WSNs) assume a vital role by continuously generating data that significantly influences the longevity and performance of the overall network. The IoT boasts an extensive array of applications^2^; however, it encounters various challenges, including security, storage, energy efficiency, and load balancing. To address these challenges in an open network with a active topologies^3^, targeted research is necessary to ensure reliable, real-time responses, energy efficiency, and fulfillment of other operational requirements for Wireless Sensor Networks (WSNs). As a data-centric network, WSNs transmit, collect, process, and store sensitive information^4^, making network security a pressing concern. WSN possess certain characteristics and limitations that make data vulnerable to tampering, damage, or theft, underscoring the need for robust network security to safeguard against various attacks^5^. While passive defense measures like firewalls and access control are valuable, they may not be adequate to counter every network attack. To address this, intrusion detection is employed as a active security measure, continuously monitoring network operations to identify and detect maloperations and attacks promptly. By doing so, the system can promptly intrude and respond as necessary to mitigate potential threats^6^.

Enhancing IoT network security against cyber threats requires the advancement of intrusion detection as an additional line of defense^7^. Various surveys have focused on machine learning (ML)-based system for intrusion detection to safeguard IoT devices from compromise. These surveys have explored intrusion detection methods for cloud-based IoT systems; WSNs, adhoc systems, and cyber security networks^8^. Indeed, conventional intrusion detection techniques face challenges in delivering sufficient and highly effective security for IoT systems, primarily due to the unique characteristics of IoT environments. These characteristics include limited bandwidth capacity^9^, energy constraints, diversity in device types and technologies, and widespread presence of IoT devices.

Machine Learning (ML) has gained credibility for successfully detecting network attacks, including those in IoT networks. However, traditional network intrusion detection models are not directly applicable to Wireless Sensor Networks (WSNs) due to their low computing and communication capabilities. Currently, researchers in the area of WSN intrusion detection are actively investigating ML models to analyze traffic data. As WSN networks expand in both scale and user base, they generate traffic data with high-dimensional characteristics, presenting challenges for conventional ML models in terms of feature extraction and detection accuracy. These limitations might not align with the specific demands of this application environment^10^. ML models, compare with traditional models, it can reduce computation burden and better learn the data traffic characteristics, leading to improved detection precision^11^.

The motivation behind proposing the novel Firefly Algorithm with Machine Learning (FA-ML) technique is rooted in the urgent necessity to fortify the security landscape of Wireless Sensor Networks integrated with the Internet of Things (WSN-IoT). This integrated system, while offering unprecedented connectivity and data exchange capabilities, is also exposed to a heightened risk of cyber threats due to its intricate and dynamic nature.

Conventional intrusion detection methods, despite their merits, face significant hurdles in effectively safeguarding IoT environments. WSNs, integral to the IoT framework, present a unique set of challenges including limited computational resources, energy constraints, device diversity, and high-dimensional traffic data. Traditional intrusion detection approaches often struggle to adapt to these distinctive characteristics, leading to suboptimal performance and leaving IoT systems vulnerable.

The FA-ML technique is driven by the vision of mitigating these vulnerabilities and advancing the state of IoT security. By commencing with data scaling, the approach ensures that the subsequent processes are aligned with the specific attributes of the data generated within WSN-IoT systems. The strategic application of the Firefly Algorithm, a nature-inspired optimization technique, for feature selection aims to enhance the efficiency and accuracy of intrusion detection. This approach is tailored to the challenges of high-dimensional data and limited resources in WSNs, a departure from conventional models that may struggle in this context.

Moreover, the integration of a Support Vector Machine (SVM) classifier, a powerful machine learning technique, with the FA-ML approach is motivated by the desire to harness the discriminative capabilities of machine learning while optimizing its performance for the unique constraints of WSN-IoT. The parameter tuning carried out by the Grey Wolf Optimizer (GWO) algorithm ensures that the SVM classifier is adeptly fine-tuned, maximizing its potential in accurately identifying intrusions.

The core motivation behind this work lies in providing a comprehensive and tailored security solution for WSN-IoT environments, mitigating the limitations of traditional intrusion detection methods. By presenting a holistic approach that not only addresses the technical intricacies but also capitalizes on the synergy between optimization algorithms and machine learning, the FA-ML technique holds the promise of significantly elevating the security posture of WSN-IoT systems. The experimental evaluation using the NSL-KDD dataset serves as empirical evidence of the technique's potential, showcasing its ability to outperform existing models and validating its viability as a potent security enhancement. Ultimately, the envisioned impact of this research is to empower IoT ecosystems with a robust, adaptable, and effective defence mechanism against an evolving landscape of cyber threats.

The paper is structured as follows: In Section “Literature review”, the related work section surveys existing intrusion detection methods for IoT, highlighting their limitations in WSN-IoT settings. The proposed Firefly Algorithm with Machine Learning (FA-ML) technique is introduced, detailing its steps in Section “Proposed Intrusion Detection System”. Results compare FA-ML with other models, demonstrating its superior intrusion detection performance in Section “Results and Discussions”. Implications and contributions to IoT security are discussed, followed by conclusions and potential future work directions Section “Conclusions”.

## Literature review

The authors in^12^ established an innovative intrusion detection system using Deep Learning (DL). They employed an optimized cluster head selection method for sensor networks, considering energy variables, distance, and delay limitations. In^13^, the Self-Improved Sea Lion Optimization (SLO) technique was introduced for the purpose of selecting the best cluster head. This approach aimed to enhance information reliability and network efficiency in an IoT system based on Wireless Sensor Networks (WSN). The research proposed both an intrusion prevention protocol and an anomalous intrusion detection protocol to bolster the overall security of the IoT-based WSN. By forming different energy-efficient groups based on node characteristics, the protocols aimed to optimize the network's performance. Additionally, in^14^, a smart system of intrusion detection for IoT-related attacks was implemented by deep learning techniques. The primary goal of this system was to identify and mitigate malicious network traffic targeting IoT devices. By employing advanced deep learning algorithms, the system sought to boost the accurateness and effectiveness of intrusion detection in the background of IoT security.

The system ensured secure IoT connectivity protocols and operational security. In^15^ introduced an enriched empirical-related component analysis for feature selection, combining PCA and empirical mode decomposition. The selected attributes were used in LSTM classification to identify attack nodes with selective attributes. These works demonstrate various approaches to improving intrusion detection in IoT and WSN networks using advanced methods and feature selection techniques.

In^16^, researchers introduced a DL-based Artificial Neural Network (ANN) method designed to achieve precise predictions of k-barrier count. This method proved beneficial for potential intrusion detection and mitigation purposes. To train and evaluate the feed-forward ANN method, four potential features were utilized: sensor sensing area, the area of the region of interest, sensing transmission area, and the number of various sensors involved. By employing these features, the ANN model was trained to accurately predict the k-barrier count, thereby contributing to improved intrusion detection and enhanced security measures.

In^17^, an intelligent system was modeled for detecting intruders in IoT-related WSNs and managing such intrusions. To develop this intelligent intrusion detection system, the author devised a feature selection technique depend on multi-objective Particle Swarm Optimization (PSO). This innovative approach aimed to optimize the selection of relevant features for intrusion detection, leveraging both rule-based criteria and multi-objective optimization provided by the PSO algorithm. By intelligently selecting the most appropriate features, the intrusion detection system could enhance its accuracy and efficiency in identifying and mitigating potential threats effectively. Additionally, an enhanced multiclass Support Vector Machine (SVM) classifier method with intelligent rules was suggested to achieve higher accuracy in detecting intruders. In^18^, a Convolutional Neural Network (CNN)-based algorithm for anomaly-based system using IoT power was presented. This algorithm demonstrated the capability to inspect all IoT traffic and effectively identify abnormal traffic behavior and potential intrusions. Indeed, the mentioned studies demonstrate the application of diverse DL and intelligent techniques for intrusion detection in IoT-related devices. By employing these advanced approaches, researchers aim to significantly enhance the accurateness and efficacy of intrusion detection and mitigation systems. The utilization of techniques such as Self-Improved Sea Lion Optimization (SLO), feed-forward ANNs, and PSO-based feature selection showcases the growing interest in developing sophisticated and effective solutions to address the security challenges prevalent in IoT environments. These innovative methods play a critical role in bolstering the overall security of IoT networks, enabling proactive measures to detect and mitigate potential intrusions, thereby safeguarding sensitive data and ensuring the seamless functioning of IoT-based systems.

In^19^, researchers introduced a hybrid method that combined a novel Convolutional Neural Network (CNN) with the Binary Chimp Optimization Algorithm (BCOA) for optimal feature classification. The BCOA was utilized to select the most relevant features in an optimal manner, enhancing the efficiency and accuracy of the subsequent classification process. Once the optimal features were identified, a fully connected CNN was trained to perform the task of classifying pixels into precise land-cover tasks. By leveraging the power of deep learning through CNNs and the feature selection capabilities of BCOA, the proposed hybrid method aimed to attain improved performance in land-cover organization tasks, ultimately enhancing the overall accuracy and reliability of the classification results.

In^20^, the author introduced a technique to enhance network parameters by combining Recurrent Neural Network (RNN) and CNN. Distinct sequences of RNN-CNN combinations were utilized for network optimization. In^21^, the author conducted an investigation into the impact of data imbalance while formulating a potential Supervisory Control and Data Acquisition (SCADA)-based intrusion detection system. SCADA systems are used for controlling and monitoring industrial processes, and ensuring their security is of utmost importance. These studies demonstrate the integration of deep learning techniques with optimization algorithms and the exploration of different neural network architectures to enhance feature selection, network parameter optimization, and intrusion detection in various applications.

In^22^, the authors proposed a novel approach called Federated-Transfer-Learning-Based Customized Distributed system (FTLCD) for identifying routing protocol intrusions in a heterogeneous IoT environment. The FTLCD approach involved a central server that initiated the process by using a predefined learning approach to construct a local model and detected unique features of several routing protocol-based IoT devices. The edge intrusion detection systems were trained using local parameters. Through federation, the central server's globally shared parameters were adapted and aggregated into various local parameters of different edge devices. The goal of this approach was to employ federated learning techniques to dynamically enhance intrusion detection capabilities across a diverse IoT environment.

In^23^, two different approaches were introduced for intrusion detection in IoT systems. The first method combined a custom CNN with Long Short-Term Memory (LSTM) deep network layers. The second approach focused on constructing an Artificial Neural Network (ANN) about each fully connected layer. Both techniques aimed to utilize the power of deep learning to enhance intrusion detection accuracy and adaptability, particularly in heterogeneous IoT environments with varying device types and protocols.

In the work presented in^24^, a novel deep learning model known as Pearson-Correlation Coefficient—Convolutional Neural Networks (PCC-CNN) is introduced. This model is designed for the purpose of identifying anomalies within networks. It achieves this by synergizing essential characteristics extracted through linear-based methods with the power of Convolutional Neural Networks. In the context of IoT networks^25^, proposes an intelligent Intrusion Detection System (IDS) that leverages deep learning techniques. The core architecture of this model involves a combination of a Recurrent Neural Network (RNN) and Gated Recurrent Units (GRU). Notably, the model showcases the ability to classify various types of attacks spanning the physical, network, and application layers. To facilitate its evaluation, this approach is trained and assessed using the ToN-IoT dataset, which is intentionally tailored for a comprehensive three-layered IoT system. This dataset is particularly valuable due to its inclusion of novel attack types that are absent in other publicly accessible datasets^26^.

Meanwhile, in^27^ introduces an innovative framework that harnesses the power of Extreme Gradient Boosting (XGBoost) machine learning, enriched through optimization with a customized variant of the Multi-Verse Optimizer metaheuristic. This framework serves as a solution for anomaly-based intrusion detection. By actively and dynamically profiling and monitoring all interconnected devices, it effectively identifies potential tampering attempts on IoT devices and detects suspicious transactions occurring within the network. In^28^ puts forth an advanced intrusion detection solution with a focus on IoT environments. This solution operates on an anomaly-based principle, constantly profiling and monitoring networked devices. Its primary aim is to uncover instances of tampering with IoT devices and to identify unusual network transactions. This solution contributes to bolstering the security of IoT ecosystems by actively identifying potential threats.

Prior methodologies for IoT intrusion detection have exhibited limitations that the proposed Firefly Algorithm with Machine Learning (FA-ML) technique aims to overcome. Existing approaches, such as DL-based systems and hybrid techniques like CNN-BCOA, often lack tailored adaptation to the unique constraints of Wireless Sensor Networks integrated with the Internet of Things (WSN-IoT). These methods might neglect resource limitations and energy constraints inherent in WSNs, leading to suboptimal performance and impracticality for real-world IoT scenarios. Additionally, while some studies focus solely on specific aspects like cluster head selection or prediction tasks, they may not offer a comprehensive intrusion detection solution for the entire IoT ecosystem. The absence of integration between optimization algorithms and classification models, as observed in several works may lead to reduced accuracy and limited effectiveness in identifying diverse intrusion types. Furthermore, the lack of empirical validation in the context of IoT environments, raises concerns about the methods' applicability and generalizability. In this regard, the FA-ML technique addresses these shortcomings by seamlessly merging the capabilities of the Firefly Algorithm with SVM classification, optimized by the Grey Wolf Optimizer. This holistic approach, tailored to WSN-IoT constraints, fills the gaps left by previous methods, providing a more adaptable, effective, and contextually relevant solution for IoT intrusion detection.

The FA-ML technique offers the integration of optimization capabilities of the Firefly Algorithm and the discriminative power of Support Vector Machine (SVM) classification. This amalgamation enables not only accurate intrusion detection but also a nuanced comprehension of the intricacies of IoT security. Moreover, the FA-ML technique is tailored to the resource constraints of WSN-IoT, ensuring efficient and adaptive performance within this unique context. Through its comprehensive integration and contextual adaptation, the FA-ML technique represents a novel and promising advancement in enhancing the security of IoT networks.

## Proposed intrusion detection system

In this article, the authors introduced a novel and automated Firefly Algorithm with Machine Learning (FA-ML) technique for achieving accurate intrusion detection in the context of WSN-IoT (Wireless Sensor Network-Internet of Things). The FA-ML technique combines the power of the Firefly Algorithm, a nature-inspired optimization algorithm, with Machine Learning methods to create an efficient and effective intrusion detection system.

By leveraging the FA-ML technique, the researchers aimed to enhance the security of WSN-IoT environments by promptly identifying and mitigating potential intrusions. The use of automation in this approach likely streamlines the intrusion detection process and contributes to precise and reliable detection, ensuring the integrity and safety of data and devices within the WSN-IoT network. Overall, this novel technique signifies a significant development in the field of intrusion detection, addressing the security challenges posed by the interconnected nature of WSNs and IoT devices.

**Figure 1:**
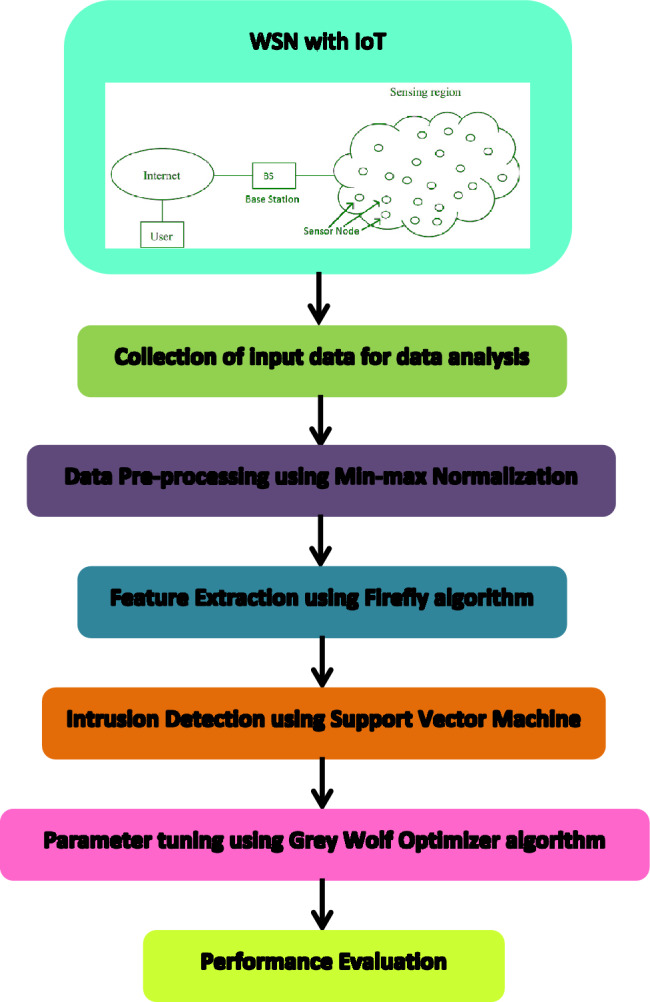
Flow chart of FA-ML technique.

The FA-ML method is specifically designed to effectively differentiate between various types of attacks, thus bolstering the complete security of the WSN-IoT system. The technique involves four main stages: data scaling, feature selection utilizing the Firefly Algorithm (FA), SVM classification, and parameter tuning using the Grey Wolf Optimizer (GWO). Figure 1 depicts the flow chart of the FA-ML technique, outlining the systematic process of intrusion detection and security enhancement in WSN-IoT.

### Data scaling

In the presented FA-ML technique, the initial stage involves data normalization. The data-scaling operation is performed to ensure that the weighted sum lies within the limit of the initiation tasks. If the data is left un-normalized, it may lead to poor training of the network and slow convergence. On the other hand, data scaling accelerates the merging and achieves dimensionless. The min–max scaling approach is employed to scale the data in the value of zero and one, and it is defined as:

1 $${Z}_{scaled}=\frac{z-{z}_{min}}{{z}_{max}-{z}_{min}}$$

where ${Z}_{scaled}$ indicates the scaled data, $z$ refers to the original value from the database, ${z}_{max}$ indicates the maximum value, and ${z}_{min}$ represents the minimum value used in the scaling process.

### Optimization of feature subset using Firefly Algorithm (FA)

Currently, the Firefly Algorithm (FA) is integrated to achieve optimal feature selection, significantly enhancing the effectiveness of intrusion detection. The FA was introduced by Xin-She Yang in 2008, drawing inspiration from the flashing behavior of fireflies in their natural environment. Fireflies emit bioluminescent light signals to attract and communicate with each other. In the algorithm, each potential solution to the optimization problem is represented as a firefly in the search region, and the brightness of each firefly corresponds to the quality of its solution. The algorithm then iteratively moves the fireflies towards brighter ones, considering their distances and attractiveness to each other. By updating the fireflies' positions depend on these directions, the algorithm efficiently explores the search region to find the optimal results. FA is particularly well-suited for complex and high-dimensional optimization tasks, converging quickly to global optima. Its easy implementation, few parameters to tune, and robustness to noisy data make it a valuable and versatile optimization tool for various real-world applications.

The Firefly Algorithm (FA) involves several major stages, which together form the optimization process:

- *Initialization*: In this stage, the initial population of fireflies is generated to represent potential solutions to the optimization problem. Each firefly's position in the search space corresponds to a candidate solution, and their brightness reflects the quality of the solution.
- Objective Function Evaluation: The objective function, which quantifies the quality of each solution, is evaluated for all fireflies. The brightness of each firefly is updated based on the objective function's values.
- *Attractiveness calculation*: The attraction of each firefly is determined based on its brightness and its distance to other fireflies. Brighter fireflies are more attractive, and fireflies that are closer to each other are more likely to move towards each other.
- *Movement of fireflies*: Fireflies move towards brighter ones, following the calculated attractiveness. The movement is guided by the attractiveness and a randomization factor to introduce exploration in the search space.
- *Updating brightness*: After the movement, the brightness of the fireflies is updated based on their new positions, reflecting the updated quality of the solutions.
- *Convergence check*: The algorithm checks for convergence, which is usually based on a predefined termination criterion, such as reaching a maximum iterations for achieving a satisfactory solution.
- *Solution selection*: The best solution among the final firefly population is selected as the optimal solution, representing the solution to the optimization problem.

These stages are iteratively repeated until the convergence criterion is met or a satisfactory solution is found. The Firefly Algorithm's ability to efficiently explore the search space, attracts towards better solutions, and converge to global optima.

The Firefly Algorithm for feature subset selection involves initializing a population of fireflies with binary solutions representing selected features. Each firefly's fitness is evaluated using a machine learning model. Fireflies are attracted to each other based on fitness and distance, guiding their movement towards more attractive solutions. Positions are updated with a movement equation that considers attraction and randomness. Light intensity, representing fitness, is updated. The process iterates for a defined number of iterations, culminating in the firefly with the highest light intensity signifying the selected feature subset optimizing a chosen performance metric. Equation components include attractiveness, movement, and light intensity updates, requiring parameter tuning for optimal performance.

The light concentration differ the following inverse square law Eq. (2),

2 $${I}_{s}=\frac{{I}_{0}}{{r}^{2}}$$

where and $r$ is the distance between the fireflies and ${I}_{0}$ represents the light concentration at the source.

The following Gaussian form Eq. (3) provides an approximation for the mutual consequence of the absorption and inverse square law.

3 $${I}_{s}={I}_{0}{e}^{-\gamma .{r}^{2}}$$

The attractiveness of the firefly is calculated by Eq. (4), where the attractiveness of firefly's is related to the light concentration observed by neighboring fireflies.

4 $${\beta }_{s}={\beta }_{0}{e}^{-\gamma .{r}^{2}}$$

where $\gamma$ is the light absorption medium coefficient and ${\beta }_{0}$ is firefly’s attractiveness at $r=0$.

Firefly’s motion is based on the principles of attractiveness: when firefly $j$ is high attractive than firefly $i$, the motion is determined by the Eq. (5)

5 $${z}_{ik}={z}_{ik}+{\beta }_{0}.{e}^{-\gamma .{r}_{ij}^{2}}. \left({z}_{ik}-{z}_{jk}\right)+ \alpha .{S}_{k}.\left[{rand}_{ik}-0.5\right]$$

where $k=0, 1, 2, \dots \dots ,D$ and $D$ is the dimension of the search area, $\alpha {\text{and}} {S}_{k}$ are the scaling functions and $rand$ is the number randomly among 0 and 1.

The distance ${(r}_{ij})$ of the fireflies $i$ and $j$ is computed using the Cartesian distance formula provided in Eq. (6).

6 $${r}_{ij}=\sqrt{\sum_{k=1}^{D}{({z}_{i,k}-{z}_{j,k})}^{2}}$$

**Figure 2:**
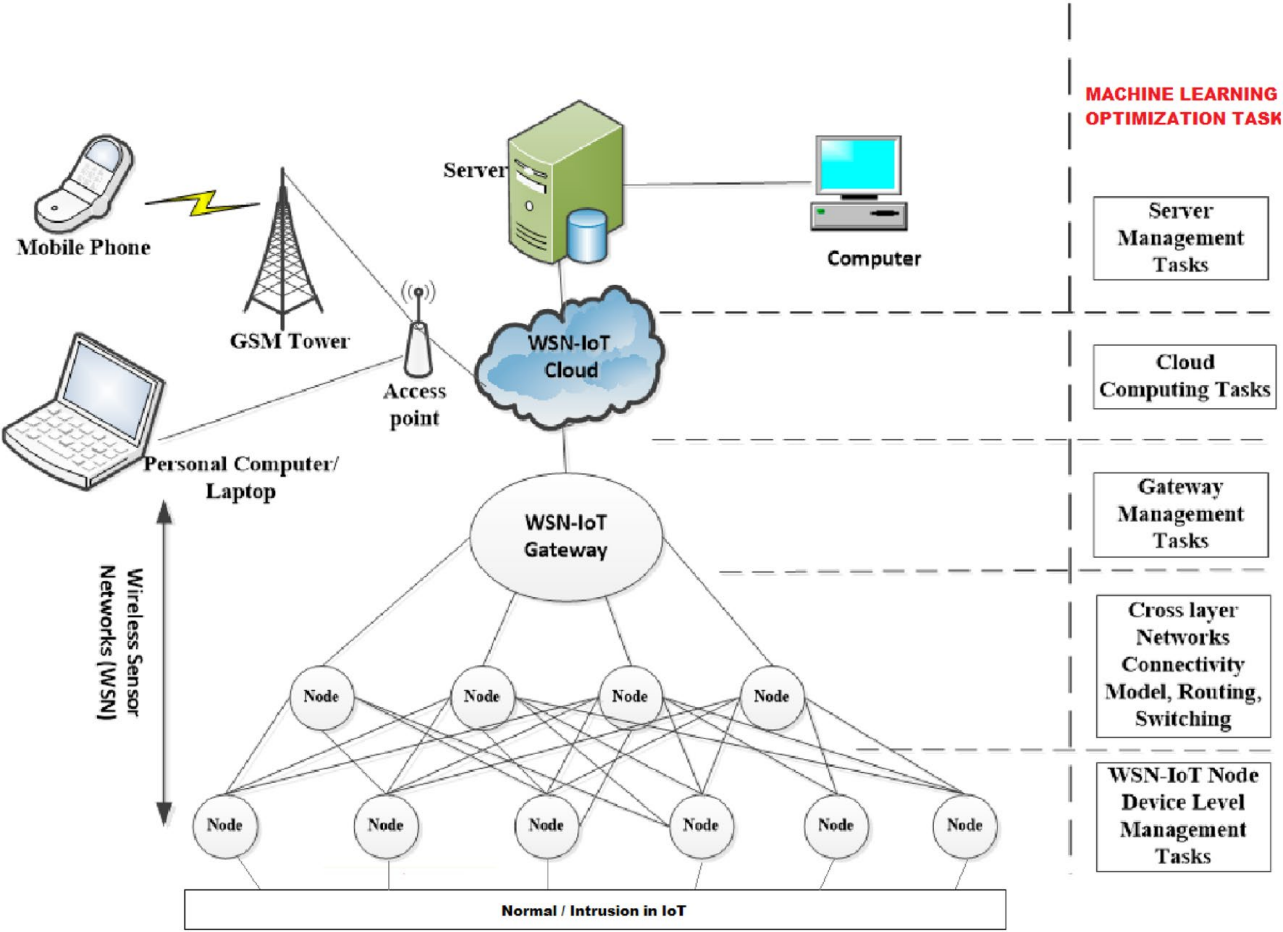
Network model of ML with WSN-IoT.

The Firefly Algorithm utilizes these formulas iteratively to guide the fireflies towards better solutions in the search space. The algorithm strikes a balance between attraction towards brighter solutions and random exploration, allowing the fireflies to efficiently explore the search space and converge towards optimal or local optima, depending on the nature of the optimization problem. This combination of attraction and exploration enables the Firefly Algorithm to effectively solve complex optimization tasks and find good solutions in various real-world applications. The network model of ML with WSN-IoT are shown in Fig. 2.

The Firefly Algorithm evaluator functions involve constructing feature subsets from fireflies' binary solutions, training machine learning models using these subsets for intrusion detection in the Wireless Sensor Network (WSN)-IoT system, calculating performance metrics like accuracy or AUC to quantify model effectiveness, assigning fitness values based on these metrics to guide the algorithm's optimization, and iteratively influencing firefly movement. This evaluation process guides the algorithm towards feature subsets that enhance intrusion detection accuracy and bolster security in the WSN-IoT.

### Intrusion detection using SVM

The FA-ML technique, employed for detecting intrusions in the WSN-IoT, utilized the Grey Wolf Optimizer (GWO) in conjunction with the Support Vector Machine (SVM) classification model within the machine learning model. The weight of a single hidden layer and input bias were randomly initialized. Subsequently, an equivalent resulting function of hidden states was calculated, considering the resultants and applying specific weights. This computation approach led to a lower computation cost for the machine learning process.

In essence, the technique involves computing an equivalent subsequent matrix of hidden layers, taking into account the resultants and applying appropriate weighted steps, which contributed to reducing the overall computation cost associated with machine learning. This indicates that the computation of the hidden states is performed efficiently, resulting in a lower computation cost for machine learning tasks. The use of a single hidden layer in neural network simplifies the network's structure and decreases the number of parameters to be trained, making it computationally efficient for certain tasks.

Let's assume we have an input vector x = [x_1_, x_2_, …, x_n_] as input to the network, and the network has M neurons in the hidden layer. To compute the output of the hidden layer, the following steps are taken:

- *Step 1*: Calculate the weighted sum of inputs for each neuron in the hidden layer: For each neuron hidden layer i (where i = 1 to M): z_i_ = Σ(w_ij_ * x_j_) + bi, where w_ij_ is the weighted sum of inputs x_j_ to the neuron at hidden layer $i$, x_j_ is the jth element of the input vector x, and b_i_ is the bias term for the ith hidden neuron.
- *Step 2*: Apply an activation function to the weighted sum for each neuron in the hidden layer: For each hidden neuron i: h_i_ = activation(z_i_), where activation( ) is a non-linear activation function, such as tanh function.
- *Step 3*: Calculate the weighted sum of the hidden layer outputs for each output neuron: For each output neuron k: y_k_ = Σ(v_ki_ * h_i_) + c_k,_ where v_ki_ is the ith weighted hidden neuron to the kth output neuron, h_i_ is the output of the ith hidden neuron, and c_k_ is the bias term for the kth output neuron.
- *Step 4*: Apply an activation function to the weighted sum for each output neuron (optional, depending on the task): For each output neuron k: y_k_ = activation(y_k_)

The calculated y_k_ signifies the output of the single hidden layer for the given input x. The weights (w_ij_ and v_ki_) and biases (b_i_ and c_k_) are parameters of the network that are learned during the training process using gradient descent technique.

In the final step, the GWO algorithm is utilized to optimally select the parameters associated with the SVM classifier. GWO is particularly effective in global optimization tasks, demonstrating its ability to efficiently explore extensive and intricate solution spaces to discover near-optimal solutions. By leveraging the GWO algorithm, the SVM classifier's parameters are tuned for optimal performance, enhancing the overall effectiveness of the intrusion detection system and leading to improved accuracy in identifying potential intrusions in the WSN-IoT environment. Moreover, the algorithm has few parameters to tune, reducing the need for extensive parameter optimization. GWO's ability to explore the search space thoroughly and converge to promising solutions has earned it recognition as an effective optimization tool in various real-world applications. Its robustness, ease of implementation, and global search capabilities make GWO a competitive choice for optimization problems across different domains. The algorithm for GWO is illustrated below:
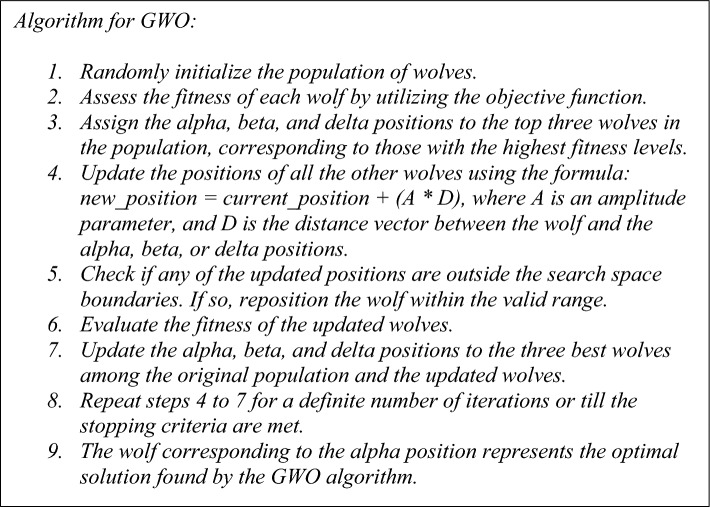


At the end of the iterations, the alpha position (α) represents the best solution found by the GWO algorithm and its fitness value fitness_j_ gives the quality of the optimal solution.

## Results and discussions

This section focuses on evaluating the performance of intrusion detection using the FA-ML method. The evaluation is carried out on the NSL-KDD dataset, consisting of 149,000 samples with two class labels as specified in Table 1.

**Table 1:** NSL-KDD Dataset.

Class	Network attacks	Number of samples	
		Training set	Test set
Legitimate traffic	Normal	67,343	10,665
Attack traffic	Denial of Service (DoS)	45,966	11,964
	User to Root (U2R)	52	228
	Probing attack (PA)	2635	5884
	Remote to Local (R2L)	1239	3024
Total no. of samples		117,235	31,765
		= 149,000	

The proposed model was implemented using the Scikit-learn tool on a PC, with the following parameter settings: learning rate: 0.01, batch size: 32, dropout: 0.2, activation function: Tanh, and epoch count: 60.

**Figure 3:**
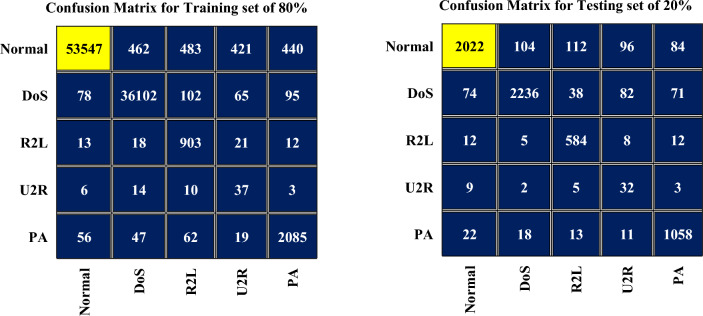
Confusion matrix for FA-ML when trained with 80% and tested with 20% of dataset.

Figure 3 illustrates the confusion matrix of the FA-ML technique, depicting different set of the training and testing dataset. The results clearly demonstrate the effective classification of both normal and attack samples achieved by the FA-ML technique. Table 2 displays the evaluation metrics of the FA-ML method achieved with 80% of the training set, while Table 3 provides the evaluation metrics with 20% of the testing set. These evaluation metrics offer valuable perceptions into the performance and efficacy of the FA-ML technique in detecting intrusions in the WSN-IoT environment.

**Table 2:** Evaluation matrices for FA-ML with 80% of training set.

Network Attacks	Accuracy (%)	Sensitivity	Specificity	F1-score	AUC score
80% of training set					
Normal	99.56	99.57	99.75	99.36	99.64
Denial of Service (DoS)	99.42	98.96	99.82	98.14	99.35
Remote to Local (R2L)	99.38	98.84	99.47	96.57	98.94
User to Root (U2R)	99.14	96.78	99.68	94.26	97.38
Probing attack (PA)	99.22	97.63	99.53	95.02	98.28
Average	99.34	98.36	99.65	96.67	98.72

**Table 3:** Evaluation matrices for FA-ML with 20% of testing set.

Network Attacks	Accuracy (%)	Sensitivity (%)	Specificity (%)	F1-score (%)	AUC score (%)
20% of testing set					
Normal	99.24	99.85	99.87	99.87	99.39
Denial of Service (DoS)	99.38	98.12	99.25	95.93	99.29
Remote to Local (R2L)	99.16	97.11	99.69	92.28	98.23
User to Root (U2R)	99.52	98.47	99.45	93.47	97.58
Probing attack (PA)	99.14	97.05	99.69	94.59	98.05
Average	99.29	98.12	99.59	96.23	98.51

**Figure 4:**
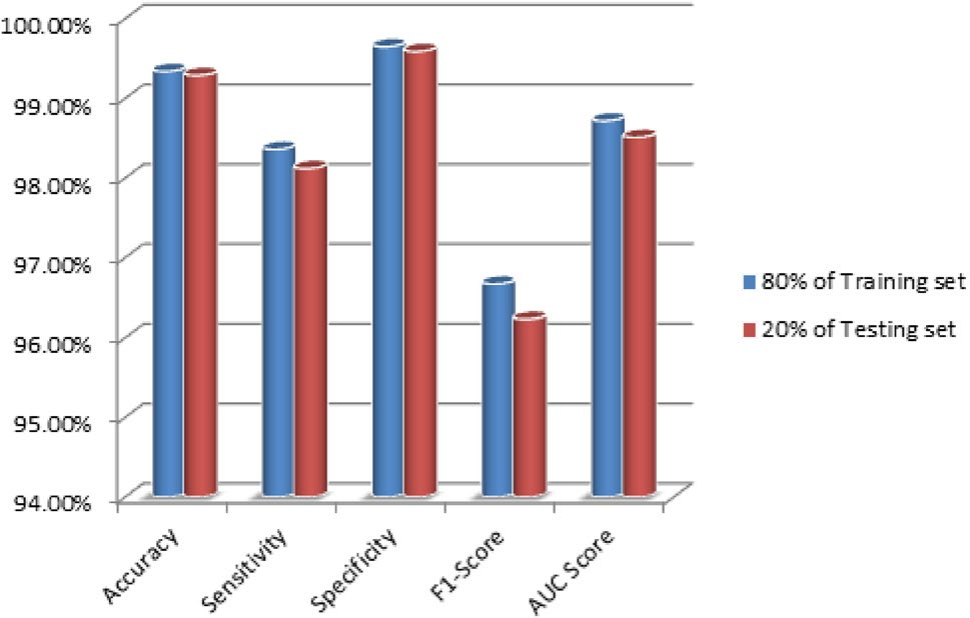
Average intrusion detection results for FA-ML under 80% of training set and 20% of testing set.

In Fig. 4, the proposed FA-ML model are depicts the results of intrusion detection when trained with 80% of the dataset and tested 20% of the dataset. The results indicate that the FA-ML technique exhibits improved performance for each individual network attack. With 80% of the training set, the FA-ML technique achieves an average accuracy of 99.34%, sensitivity of 98.36%, specificity of 99.65%, F1-score of 96.67%, and AUC score of 98.72%. Similarly, when tested with 20% of the dataset, the FA-ML approach attains an average accuracy of 99.29%, sensitivity of 98.12%, specificity of 99.59%, F1-score of 96.23%, and AUC score of 98.51%. These evaluation metrics demonstrate the reliability and efficacy of the FA-ML technique in detecting intrusions in the WSN-IoT environment, as it consistently performs well under various network attack scenarios.

**Figure 5:**
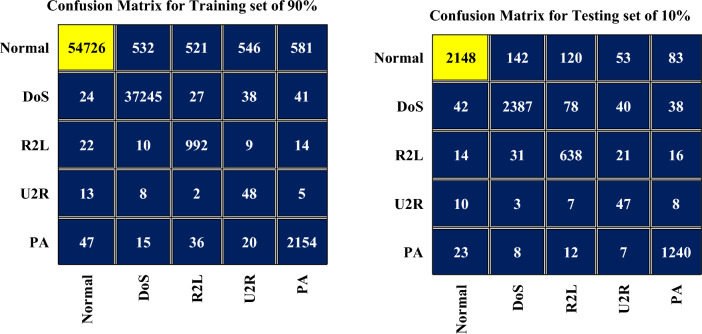
Confusion matrix for FA-ML with 90% of training set and 10% of testing set.

In Fig. 5, the confusion matrices of the FA-ML technique are presented, depicting its performance under different set of the training (90%) and the testing (10%). The figures illustrate the effective classification of attacks and normal samples achieved by the FA-ML technique. Table 4 displays the evaluation metrics of the FA-ML technique when trained with 90% of the dataset, providing valuable insights into its performance. Similarly, Table 5 presents the evaluation metrics obtained with 10% of the testing set, offering a comprehensive assessment of the FA-ML technique's ability to detect intrusions.

**Table 4:** Evaluation matrices for FA-ML with 90% of training set.

Network Attacks	Accuracy (%)	Sensitivity (%)	Specificity (%)	F1-score (%)	AUC score (%)
90% of training set					
Normal	99.66	99.68	99.83	99.84	99.85
Denial of Service (DoS)	99.54	99.12	99.96	98.64	99.96
Remote to Local (R2L)	99.47	98.97	99.76	96.46	99.10
User to Root (U2R)	99.85	97.58	99.84	94.75	98.24
Probing attack (PA)	99.69	98.38	99.92	96.14	98.86
Average	99.64	98.75	99.86	97.17	99.20

**Table 5:** Evaluation matrices for FA-ML with 10% of testing set.

Network attacks	Accuracy (%)	Sensitivity (%)	Specificity (%)	F1-score (%)	AUC score (%)
10% of testing set					
Normal	99.47	99.87	99.89	99.91	99.69
Denial of Service (DoS)	99.58	98.48	99.44	96.78	99.87
Remote to Local (R2L)	99.69	97.82	99.72	95.85	98.75
User to Root (U2R)	99.28	98.52	99.68	94.58	98.58
Probing attack (PA)	99.36	97.21	99.76	95.58	98.86
Average	99.48	98.38	99.70	96.94	99.15

**Figure 6:**
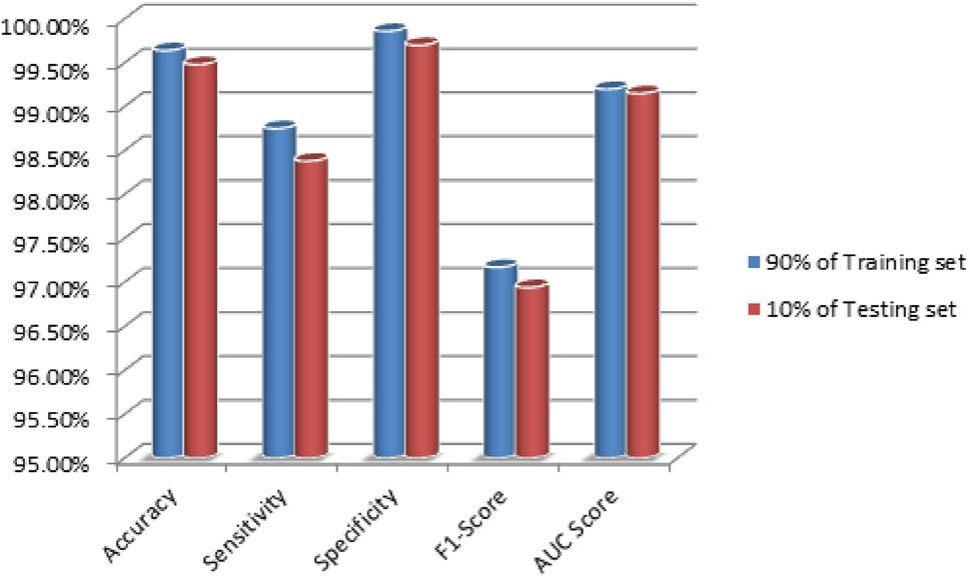
Average intrusion detection results for FA-ML under 90% of training set and 10% of testing set.

In Fig. 6, the proposed FA-ML model are displayed when trained with 90% of the dataset and tested with the remaining 10% to depict the results of intrusion detection. The results reveal that the FA-ML technique achieves improved performance for each individual network attack. With 90% of the training set, the FA-ML technique attains an average accuracy of 99.34%, sensitivity of 98.36%, specificity of 99.65%, F1-score of 96.67%, and AUC score of 98.72%. Similarly, when tested with 10% of the dataset, the FA-ML approach demonstrates an average accuracy of 99.29%, sensitivity of 98.12%, specificity of 99.59%, F1-score of 96.23%, and AUC score of 98.51%. These evaluation metrics further underscore the effectiveness and reliability of the FA-ML method in detecting intrusions in the WSN-IoT environment, as it consistently performs well across various network attack scenarios and different training and testing set sizes.

**Figure 7:**
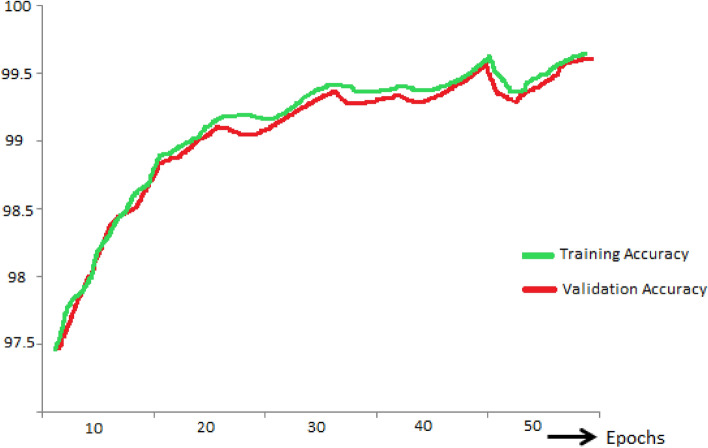
Training accuracy and Validation accuracy of FA-ML model.

In Fig. 7, the training accuracy (ACC_Tra_) and validation accuracy (ACC_Val_) of the FA-ML model were examined to explore the performance of intrusion detection in the WSN-IoT environment. The figure demonstrates that as the values of ACC_Tra_ and ACC_Val_ increase, the FA-ML model exhibits improved performance. In particular, the FA-ML method achieves its highest performance with maximum ACC_Tra_ values, indicating strong training accuracy. This suggests that the FA-ML model effectively learns and fits the training data, leading to enhanced detection capabilities in identifying intrusions within the WSN-IoT system. The increasing trend of ACC_Val_ indicates that the model also generalizes well to unseen data during the validation phase, further validating its efficacy in intrusion detection.

**Figure 8:**
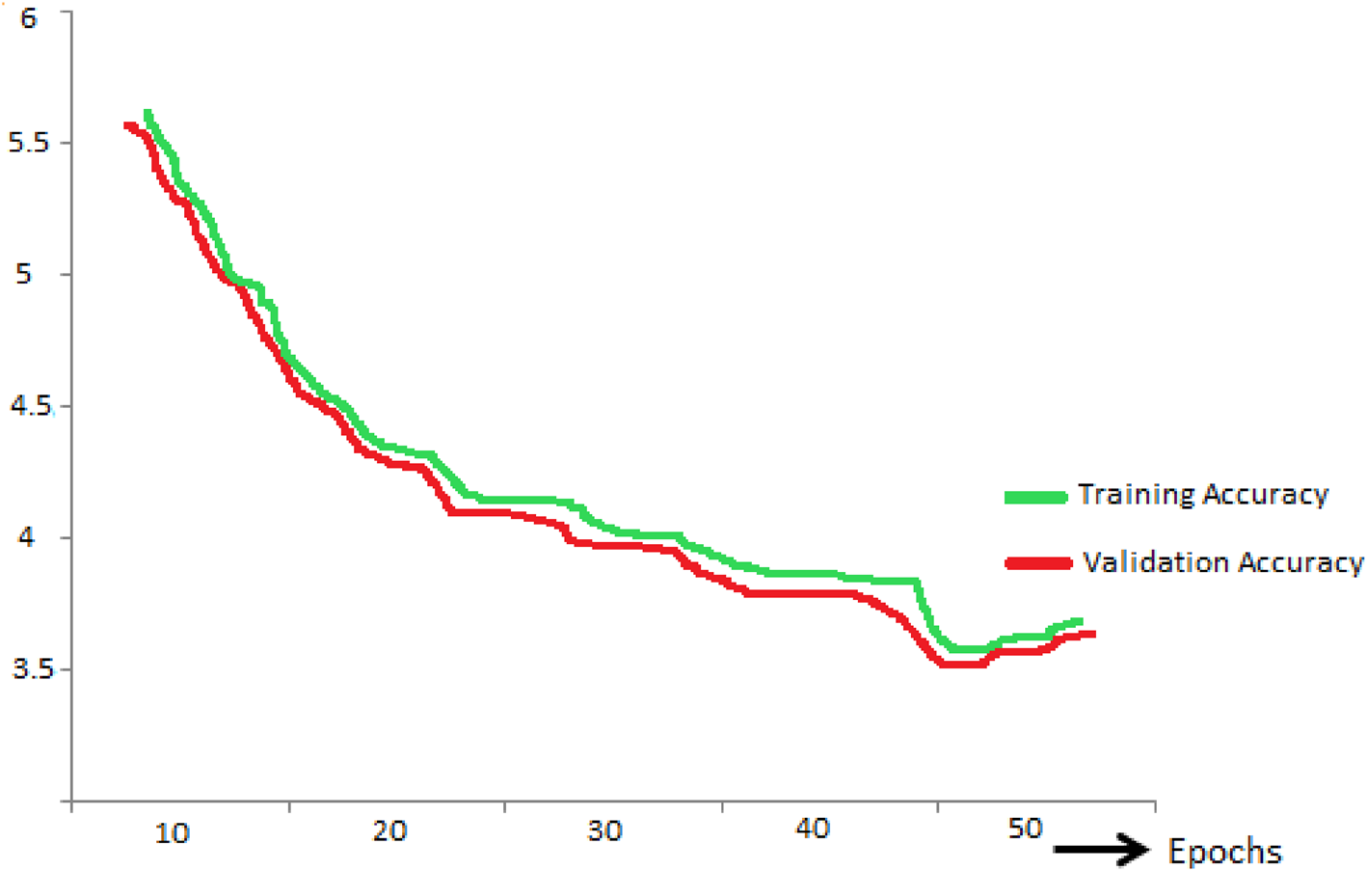
Training loss and Validation loss of FA-ML model.

In Fig. 8, the training loss (Loss_Tra_) and validation loss (Loss_Val_) of the FA-ML approach were evaluated to assess the performance of intrusion detection in the WSN-IoT environment. The figure demonstrates that the FA-ML method achieves greater performance with minimal of Loss_Tra_ and Loss_Val_. The decreasing trend of Loss_Tra_ indicates that the FA-ML model effectively minimizes the training loss during the learning process, leading to improved convergence and effective learning from the training data. Similarly, the decreasing values of Loss_Val_ suggest that the model generalizes well to unseen data during the validation phase, resulting in a reduction in validation loss.

Overall, the FA-ML model demonstrates excellent performance by consistently achieving lower values of both training and validation loss, further reinforcing its efficacy in intrusion detection for WSN-IoT applications.

**Figure 9:**
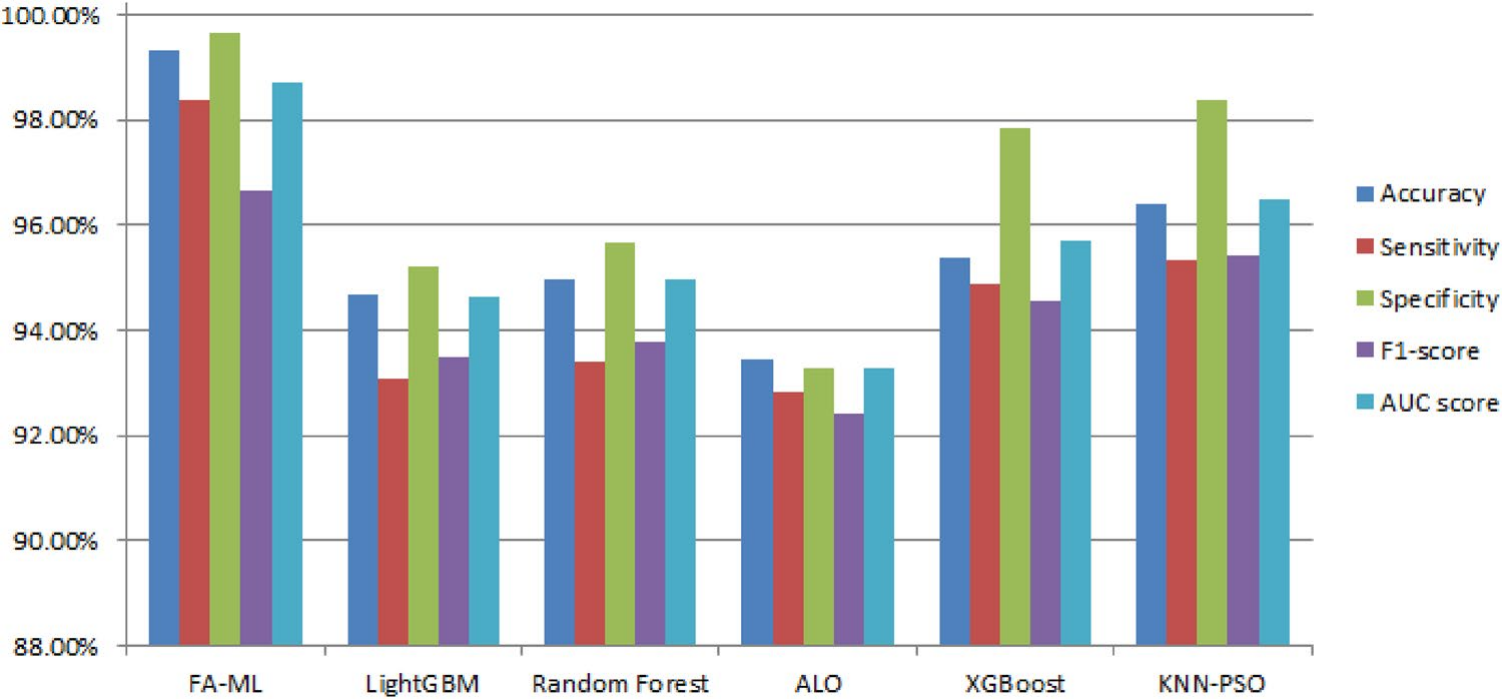
Comparative results of FA-ML with other techniques.

Table 6 and Fig. 9 present the classification results of the FA-ML model in comparison with other algorithms like LightGBM, Random forest, Particle Swarm Optimization with K-Nearest Neighbors (KNN-PSO), Ant Lion Optimizer (ALO), and XGBoost. The performance of these models is estimated based on various metrics. The outcomes reveal that LightGBM, Random forest, and ALO algorithms exhibit the poorest performance compared to the other models. XGBoost demonstrates moderately improved results, while the KNN-PSO model shows considerably better performance with an accuracy of 96.42%, sensitivity of 95.35%, specificity of 98.36%, F1-score of 95.42%, and AUC score of 96.48%. However, the FA-ML technique outperforms all other models, achieving a maximum performance with an accuracy of 99.34%, sensitivity of 98.36%, specificity of 99.65%, F1-score of 96.67%, and AUC score of 98.72%. These impressive results indicate the superiority of the FA-ML technique in accurately classifying and detecting intrusions, making it a highly effective approach for intrusion detection in WSN-IoT systems.

**Table 6:** Performance analysis of FA-ML with other techniques under 80% of training set and 20% of testing set.

Techniques	Accuracy (%)	Sensitivity (%)	Specificity (%)	F1-score (%)	AUC score (%)
FA-ML	99.34	98.36	99.65	96.67	98.72
LightGBM	94.67	93.08	95.22	93.48	94.65
Random Forest	94.98	93.41	95.68	93.78	94.98
ALO	93.46	92.85	93.27	92.42	93.28
XGBoost	95.36	94.87	97.85	94.57	95.69
KNN-PSO	96.42	95.35	98.36	95.42	96.48

**Figure 10:**
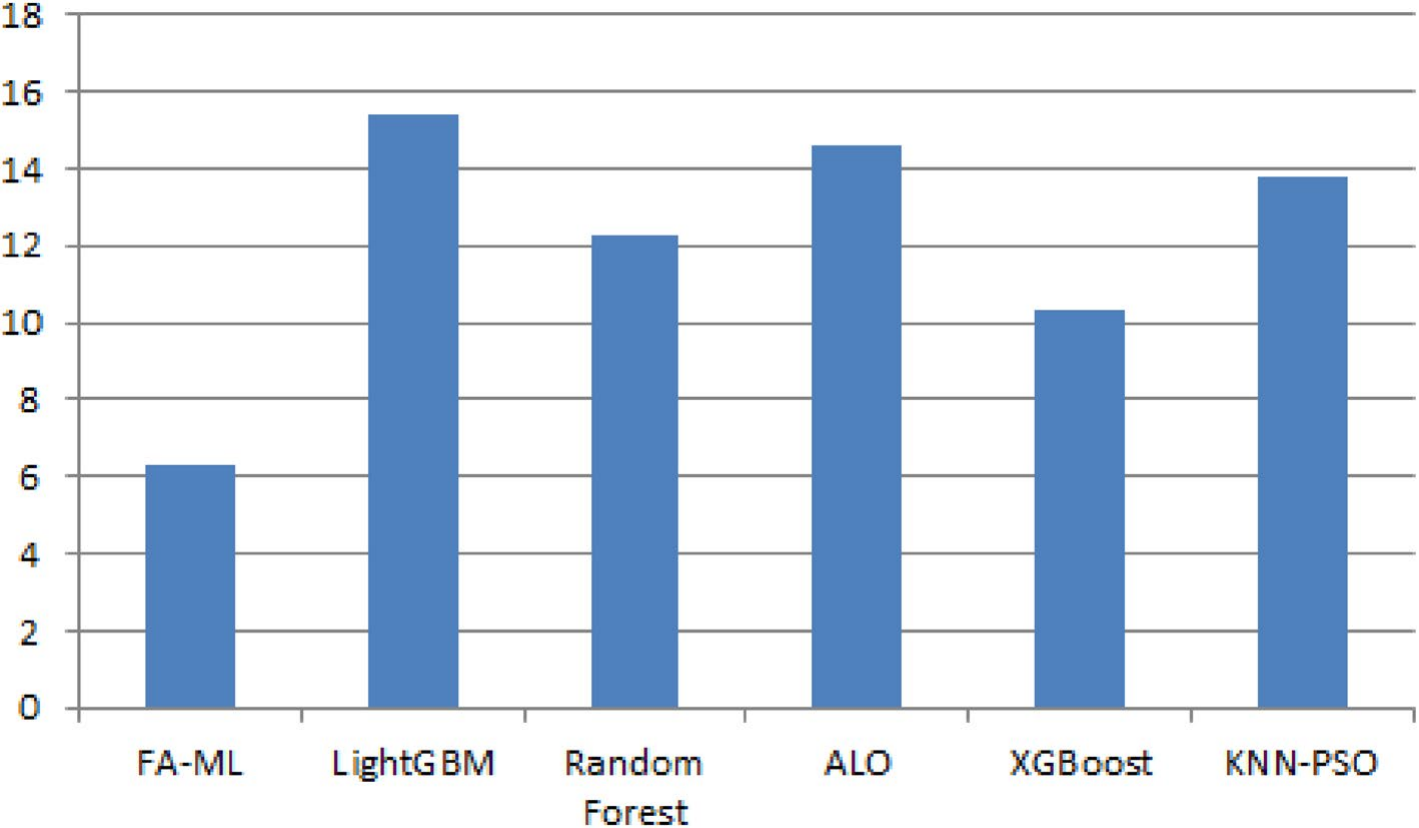
Computation time of FA-ML with other techniques in seconds.

Table 7 and Fig. 10 present the computation time results of the FA-ML method in comparison to existing methods. The study examines the computational efficiency of LightGBM, Ant Lion Optimizer (ALO), and Particle Swarm Optimization with K-Nearest Neighbors (KNN-PSO) algorithms. The experimental outcomes reveal that these algorithms result in ineffectual performance with higher computational times compared to other models. Random Forest attempts to demonstrate slightly reduced computational time, while the XGBoost model shows somewhat considerable performance with a computational time of 10.3 s. On the other hand, the FA-ML technique outperforms the other models in terms of computational efficiency, with a lower computational time of 6.3 s. These results provide strong evidence supporting the improved detection performance of the FA-ML method within the WSN-IoT environment. The incorporation of FA for feature subset selection and the utilization of the GWO algorithm for parameter tuning have significantly contributed to the improved performance of the proposed model. This combined approach makes the FA-ML technique a more efficient and effective solution for intrusion detection in WSN-IoT systems, demonstrating its potential to outperform existing methods in relation to computational efficiency and accuracy.

**Table 7:** Computation time of FA-ML with other techniques.

Techniques	Computational time (sec)
FA-ML	6.3
LightGBM	15.4
Random Forest	12.3
ALO	14.6
XGBoost	10.3
KNN-PSO	13.8

## Conclusions

This research introduces an automated FA-ML method designed to achieve precise intrusion detection, thereby enhancing security in WSN-IoT systems. The proposed FA-ML technique follows a systematic approach, including data scaling, FA-based feature selection, SVM classification, and GWO based parameter tuning, to effectively identify intrusions. The experimental evaluation of the FA-ML technique on the NSL-KDD intrusion dataset demonstrated remarkable results, achieving a extreme accuracy of 99.34%. Future studies can potentially enhance the effectiveness of the suggested technique by incorporating WSN intrusion detection models under semi-supervised or unsupervised conditions. By integrating these models, the detection capabilities can be expanded to include attacks, like sybil attacks, routing attacks, and other potential intrusions, further strengthening the security of WSN-IoT systems. By integrating these advanced detection techniques, the FA-ML technique can be further strengthened to provide robust security for WSN-IoT systems, addressing a wider range of potential threats and vulnerabilities.

In future, the proposed FA-ML method's efficacy in precise intrusion detection within WSN-IoT systems can be further enhanced by delving into semi-supervised or unsupervised learning for tackling novel attack types. Specifically, more targeted detection approaches could be developed for sybil attacks, routing attacks, and other specific intrusions, while exploring ensemble techniques like random forests and stacking could bolster overall detection robustness. Dynamic adaptation mechanisms need to be researched to enable the FA-ML model to adjust to evolving threats and changing network dynamics. Additionally, refining feature selection through alternative methods, fortifying the model against adversarial attacks, and testing it with real-world data from WSN-IoT deployments are crucial steps. Optimization for resource-constrained environments, interpretability enhancements, and collaborative defense mechanisms are further avenues for strengthening the FA-ML technique's security capabilities across a broader spectrum of threats in WSN-IoT systems.
